# Managers’ Micro-Communities Matter: The Impact of Clinical Supervision Team on Therapist Perception of the Organization

**DOI:** 10.1007/s10488-024-01376-0

**Published:** 2024-04-27

**Authors:** Meredith R. Boyd, Kimberly D. Becker, Alayna L. Park, Kaitlyn Pham, Bruce F. Chorpita

**Affiliations:** 1 Department of Psychology, University of California Los Angeles, 502 Portola Plaza, Los Angeles, CA 90095 USA; 2 Department of Psychology, University of South Carolina, Columbia, SC USA; 3 Department of Psychology, University of Oregon, Eugene, OR USA

**Keywords:** Organizational climate, Clinical supervision, School-based mental health, Middle manager

## Abstract

Positive organizational climate — employee perceptions of their work environment and the impact of this environment on well-being and functioning — is associated with desirable organizational and client-level outcomes in mental health organizations. Clinical supervisors are well-positioned to impact organizational climate, as they serve as intermediaries between higher-level administrators who drive the policies and procedures and the therapists impacted by such decisions. This cross-sectional study examined the role of clinical supervisors as drivers of therapist perceptions of organizational climate within supervisory teams. Specifically, the present study investigated: (1) shared perceptions of organizational climate among therapists on the same supervisory team; (2) predictors of therapist climate perceptions. Eighty-six therapists were supervised by 22 supervisors. Indices of interrater agreement and interrater reliability of therapists on the same supervisory team were examined to determine shared or distinct perceptions of organizational climate. Multi-level models were used to examine whether supervisor attitudes towards evidence-based practices and therapist perceptions of supervisor communication predicted perceived organizational climate. Results showed perceptions of organizational cohesion and autonomy were shared among therapists on the same supervisory team and distinct from therapists on different supervisory teams. Therapist perceptions of their supervisor’s communication was positively associated with perceptions of organizational cohesion and autonomy. These findings align with emerging evidence that middle managers shape their employees’ experience of their work environment through communication strategies. These findings also point to the potential for intervening at lower organizational levels to improve overall organizational climate.

## Introduction

There is a substantial evidence base of interventions to treat child and adolescent mental health conditions (Chorpita et al., 2011). However, interventions supported by research remain challenging to implement and underused in community mental health settings where most children and families access services (Peters-Corbett et al., 2023; Borntrager et al., 2013; Garland et al., 2010). Differences in the organizational contexts in which evidence-based treatments (EBTs) are developed versus implemented has been hypothesized as contributing to the gap between what is established in the evidence base and what is done in the therapy room (Peters-Corbett et al., 2023; Chorpita et al., 2014; Hoagwood et al., 2001; Southam-Gerow et al., 2008). For example, therapists in community mental health settings experience significant strain and competing demands within their organizations distinct from therapists who participate in efficacy trials. Specifically, therapists in efficacy trials are often affiliated with high-resource research institutes, treat a small number of clients, and receive a high degree of support and supervision (Hoagwood et al., 2001). In contrast, community-based therapists often carry large clinical caseloads (e.g., Dorsey et al., 2017; Lau et al., 2018) and treat high acuity clients with greater comorbidity and adverse life circumstances compared with clients included in efficacy trials (Chorpita et al., 2014; Southam-Gerow et al., 2008).

## Organizational Climate

Acknowledging the possible impacts of organizational characteristics on the quality of care delivered, organizational climate has gained attention as a determinant that may hinder or facilitate the implementation of EBTs in community mental health settings. Although there are several definitions in the literature, *organizational climate* is commonly defined as employee perceptions of their work environment and the impact of this environment on well-being and functioning (Glisson & James, 2002; Ouellette et al., 2020). Positive organizational climate is associated with a number of desirable outcomes including improved therapist attitudes towards their work and EBTs (e.g., Aarons & Sawitzky, 2006; Aarons & Sommerfeld, 2012; Brimhall et al., 2016), improved EBT quality and fidelity (e.g., Olin et al., 2014; Williams et al., 2019), increased client engagement and satisfaction with services (e.g., Greener et al., 2007; Lehman et al., 2002), and improved clinical outcomes (e.g., Glisson & Green, 2011). Conversely, poor organizational climate is associated with therapist burnout and organizational turnover (Aarons & Sawitzky, 2006; Glisson et al., 2008). For example, Glisson et al. (2008) generated profiles of organizational climates for clinics across the United States and found yearly turnover rates of organizations with the most negative climates were double the rates of organizations with the most positive climates. Such findings emphasize the importance of better understanding drivers of organizational climate.

## Levels of Analysis

Within the mental health service literature, investigations of organizational climate tend to focus on individual therapist perceptions rather than considering organizational units (for exception see work from Glisson and colleagues such as Glisson & James, 2002), potentially missing useful insights that could be gained through examination of various organizational levels. In the broader organization management and leadership literature, there is a longer history of exploring how various organizational units (e.g., hospitals, army bases, warehouses) and sub-units (e.g., clinical teams, platoons, shift managers) impact critical organizational outcomes (e.g., turnover, ethical behavior, accuracy in medication administration; Chan, 2014; Zohar & Luria, 2005). 

Middle managers (i.e., front-line leaders such as employees’ direct supervisors) reflect an organizational level that may influence climate in mental health organizations (Birken et al., 2012, 2018). In community mental health settings, it is common for clinical supervisors to oversee the practice of therapists (Schoenwald et al., 2008). Clinical supervisors thus represent middle managers within mental health systems. They are well-positioned to positively impact organizational climate as they work directly with therapists who deliver treatment and serve as intermediaries between higher level administrators who dictate the policies and procedures and the therapists impacted by such decisions. Indeed, in their study of the activities performed by supervisors during supervision, Bailin et al. (2018) found that clinical supervisors spend time both on administrative tasks (i.e., implementation of policies and procedures) as well as supporting therapist clinical practice (e.g., case conceptualization, recommendation of practices, and modeling skills).

There is growing evidence of the influence of supervisors in community mental health organizations (Aarons et al., 2011; Brimhall et al., 2016; Green et al., 2014). For example, Aarons et al. (2011) found that therapists’ perceptions of the leadership style of their direct supervisor was associated with attitudes towards use of evidence-based practices and Aarons et al. (2021) found that therapists’ perception of their own relationships with their supervisor compared with other team members’ relationships with the same supervisor was associated with intention to leave the organization. Bunger et al.’s (2019) qualitative study sheds light on possible avenues through which supervisors influence therapist attitudes. Specifically, in their study of therapists in a child welfare system undergoing organizational changes, Bunger et al. (2019) found that supervisors shaped therapist perceptions of organizational climate through their communication about the novel intervention, day-to-day support using the intervention, and persuasion about the value of the intervention. Finally, in their study of clinical and case management therapists and their direct supervisors in community mental health agencies in San Diego County, Green et al. (2014) found that strong leadership was associated with positive organizational climate which was, in turn, associated with strong working alliance between therapists and their clients, demonstrating a through line from supervisor to the therapy room.

However, in the reviewed studies, analyses were conducted by nesting therapists within organizations rather than within supervisors (for exception see Aarons et al., 2021), making it difficult to determine the extent to which therapist attitudes and perceptions were influenced by supervisor rather than by other aspects of the organization. Because therapists were asked to react to leadership specifically, it can be inferred that supervisors impacted therapist perceptions. An alternative explanation is that additional, unaccounted for organizational variables may have influenced the way therapists experienced the leadership of their supervisor. An understanding of the potential impact of the supervisor on therapist perceptions of organizational climate could inform additional interventions to improve climate and consequently reduce organizational turnover and enhance the quality of care delivered. If it is found that supervisors influence therapist perceptions of organizational climate, then mental health organizations might prioritize training for supervisors to increase their skill and competence as middle managers or organizations might protect additional time in the workday for supervisors to engage in tasks that support their supervisory team. Organizations might also monitor the organizational climate of supervisory teams and use this information to provide targeted support to supervisors of teams with poorer climates.

## Study Aims

The overarching goal of the present study was to better understand the role of clinical supervisors as potential drivers of therapist perceptions of organizational climate. The aims of this study were (1) to determine if perceptions of organizational climate were shared among therapists in the same supervisory teams and distinct from therapists in different teams, and (2) to investigate candidate, supervisor predictors of therapist perceptions of climate. Regarding the first aim, it was hypothesized that therapists in the same supervisory team would have greater shared perceptions of organizational climate compared with therapists in different supervisory teams as indicated by two interrater agreement parameters (i.e., a_wg_ and r_wg(j)_) and interrater reliability (intraclass correlation coefficient, ICC; Schneider et al., 2012) calculated from multilevel models of therapists nested within supervisory teams. Regarding the second aim, it was hypothesized that supervisor attitudes toward evidence-based practices and therapist perceptions of supervisor communication would be significantly and positively associated with therapists’ perceptions of organizational climate. Previous literature has shown that therapists’ own attitudes towards evidence-based practices is associated with their experience of their organization (Aarons & Sawitzky, 2006; Aarons & Sommerfeld, 2012) and their experience of their direct supervisor mediated by organizational climate (Brimhall et al., 2016). Because supervisors have greater organizational power and influence, it was anticipated that supervisor attitudes would serve as a driver of organizational climate. With this reasoning in mind, supervisor attitudes towards evidence-based practices was selected as a candidate predictor. Supervisor communication was selected as a candidate predictor given the role of middle managers in bridging communication from upper management to direct reports and thus shaping perceptions of organizational climate (Bailin et al., 2018; Bunger et al., 2019).

## Methods

### Participants and Setting

Participants for the present study were recruited as part of a multi-site cluster randomized trial examining the impact of a coordinated knowledge system on therapist use of evidence and subsequent client engagement in school-based mental health services (Chorpita & Becker, 2017–2022). Study procedures were approved by the Institutional Review Boards (IRB) at the University of South Carolina and University of California, Los Angeles as well as IRBs of participating organizations that requested independent review. Written informed consent was obtained from study participants and study procedures were carried out in accordance with local IRB requirements. The present study was not preregistered.

Supervisors and therapists were employed by either the Los Angeles Unified School District School Mental Health and Wellness Center Program in urban Los Angeles, California (CA; therapists *n* = 45, supervisors *n* = 12) or by the South Carolina Department of Mental Health in rural South Carolina (SC; i.e., Pee Dee and Santee-Wateree catchment areas; Pee Dee therapists *n* = 19, supervisors *n* = 4; Santee-Wateree, therapists *n* = 22, supervisors *n* = 6). The Los Angeles Unified School District is a mental health services subsidiary in which the school district directly employs therapists who work in integrated school mental health clinics. By contrast, in South Carolina, therapists, who are employed by the Department of Mental Health, deliver services to schools through contracts between school districts and regional centers. For qualitative information about the ways in which therapists interfaced with the schools in which they served, see Lakind et al. (2023). For information about the frequency, length and content of supervision see Chorpita et al. (under review) or Becker and Chorpita (2023).

Due to staff schedules and availability in CA, some supervisory dyads were created for the purpose of the trial and did not exist prior. Supervisory dyad creation did not occur in SC. Supervisors (*n* = 22) and therapists (*n* = 86) were included in the present study if they completed the main measure of interest (Texas Christian University Organizational Climate Scales, TCU-ORG; Lehman et al., 2002) and were part of pre-trial supervision dyads (i.e., not a supervision dyad created for the purpose of trial participation). Supervisors and therapists were predominantly master’s-level (supervisors 100%, therapists 98%) and women (supervisors 95%, therapists 90%). Supervisors were 50% Black/African American, 18% Hispanic/Latinx, 18% White/Caucasian. Therapists were 42% Black/African American, 39.53% Hispanic/Latinx and 15% White/Caucasian. See Table 1 for full demographic information for the study sample.

**Table 1:** Demographics of Supervisor and Therapists

	Supervisors (*n* = 22)		Therapists (*n* = 86)		Total (*n* = 108)	
	*N*	%	*N*	%	*N*	%
*Race/Ethnicity*						
Asian	1	4.55	2	2.33	3	2.78
Black/African American	11	50.00	36	41.86	47	43.52
White/Caucasian	4	18.18	13	15.12	17	15.74
Hispanic/Latino	4	18.18	34	39.53	38	35.19
Multiracial	2	9.09	1	1.16	3	2.78
Gender*						
Woman	21	95.45	78	90.70	99	91.67
Man	1	4.55	8	9.30	9	8.33
*Site*						
Los Angeles	12	54.55	45	52.33	57	52.78
Santee Wateree	6	27.27	22	25.58	28	25.93
Pee Dee	4	18.18	19	22.09	23	21.30
*Academic Degree*						
Bachelor's-level	0	0.00	1	1.16	1	0.94
Master's-level	20	100.00	84	97.67	104	98.11
PhD	0	0.00	1	1.16	1	0.94
*Licensed*			50	58.14		
	*M*	*SD*	*M*	*SD*	*M*	*SD*
Age	47.15	10.53	38.60	10.15	40.42	10.77
Years of Clinical Experience	15.83	8.09	6.73	5.85	8.44	7.24

^*^No participants identified as non-binary, transgender, or other (with the option to specify). Two supervisors did not provide years of experience or degree type. Eight therapists did not provide age

Excluded participants (i.e., those who did not complete the TCU-ORG and/or were not part of pre-trial supervision dyad; *n* = 58) were compared to included participants (*n* = 108) across demographic variables to determine if there were statistically significant differences that might suggest bias in results. Welch’s T-tests did not reveal differences in years of clinical experience and age. Chi-Square Goodness of Fit Tests did not reveal differences in the proportion of White or Latinx therapists in the excluded versus included participant samples. Across the full sample, the number of Asian therapists was too small for Chi-Square testing (included *n* = 4; excluded *n* = 4). The proportion of Black therapists in the excluded versus included sample was significantly different (*x*^*2*^(1, 166) = 5.53, *p* = 0.02) such that the excluded participants had a smaller proportion of Black therapists (22%) compared with the included participants (56%). In addition, the proportion of SC therapists in the excluded versus included sample was significantly different (*x*^*2*^(1, 166) = 16.44, *p* < 0.001) such that the excluded participants had a smaller proportion of SC therapists (13%) compared with the included participants (47%). As stated previously, trial-created dyads (which occurred in CA only) were excluded from the present analyses resulting in a lower proportion of CA therapists in the included sample. Exclusion of these supervisory dyads also resulted in a higher proportion of Black therapists in the included sample as SC had more Black therapists compared with CA. Across the full sample, the number of therapists who were men was too small for Chi-Square testing (included *n* = 9; excluded *n* = 2).

### Measures

#### Climate

Therapists completed the Texas Christian University Organizational Climate Scales (TCU-ORG; Lehman et al., 2002) between August 2018 and February 2020 as part of a battery of surveys delivered during study training events, site visits to collect data, and follow up with individual providers. The TCU-ORG is comprised of 30 self-report items that assess the following domains of organizational climate: clarity of organizational mission (e.g., “Your program operates with clear goals and objectives”; subscale labeled, “Mission”), cohesion among staff members (e.g., “Staff members at your program work together as a team”; subscale labeled “Cohesion”), autonomy when making decisions (e.g., “Counselors in your program are given broad authority in treating their clients”; subscale labeled “Autonomy”), communication (e.g., “The formal and informal communication channels in your program work very well”; subscale labeled “Communication”), stress (e.g., “Staff members at your program often show signs of high stress and strain”; subscale labeled “Stress”), and attitudes toward change (e.g., “The general attitude in your program is to accept new and changing technology”; subscale labeled “Change”). Each item was scored on a four-point scale from 1 (strongly disagree) to 4 (strongly agree). Consistent with the scoring instructions for this measure (Institute of Behavioral Research, 2005), select items were reverse scored across the Mission, Cohesion, Autonomy, Communication, and Change subscale. Scores for each subscale were calculated by taking the mean for the number of completed items resulting in scores that could range from 1 to 4 for each subscale with high scores reflecting strong climate except for the Stress subscale. Subscales were removed listwise if more than 50% of items were missing in the given subscale. In the current data set, 12% (*n* = 10) of participants had some missing data but only one participant was missing more than 50% of the items for one subscale. A total climate score was generated by additionally reverse scoring the items on the Stress subscale and taking the mean for the number of completed items across all subscales.

The wording of the TCU-ORG aligns with Schneider’s (2013) definition of climate as *shared* perceptions about the *collective* impact of work environment given that respondents were asked to reflect on how they perceive the organization impacting other staff members (e.g., “*staff members* at your program often show signs of high stress and strain” rather than, “*I feel* a high degree of stress and strain). To reinforce the referent of the survey as the broader organization, the following instruction was included at the top of the survey in bold: “For the following 30 questions, please consider the School Mental Health Program.” Referent-shift consensus items refer to items that are worded such to prompt respondents to answer questions in relation to the same level of aggregation, in this specific case to answer questions in relation to the organization to which they belong rather than another level of grouping such as the school system as a whole or the individual school in which they work (Chan, 1998). In Lehman et al.’s (2002) study, reliability estimates for six of the seven subscales were above 0.70 except for the Autonomy subscale (*a* = *0.5*7). In the present study the reliability estimates were as follows: Change (*a* = 0.64), Cohesion (*a* = 0.87), Communication (*a* = 0.84), Mission (*a* = 0.70), Stress (*a* = 0.81), Autonomy (*a* = 0.39), and Total Score (*a* = 0.84). See Table 2 for supervisory team level descriptive statistics for this measure.

**Table 2:** Supervisory Team Descriptive Statistics, Intraclass Correlation Coefficients, and Reliability Estimates for Climate Subscales and Total Score

Scale Name	*M (SD)*	Range	ICC (1)	ICC (2)	r_wg(j)_ *M*	r_wg(j)_ Range	a_wg_ *M*	a_wg_ Range
Autonomy	2.68 (.25)	2.16 to 3.00	**0.38**	0.66	0.90	.30 to .98	0.74	.22 to .92
Change	2.73 (.24)	2.20 to 3.07	0.10	0.28	0.88	.00 to .99	0.73	.08 to .96
Cohesion	2.92 (.35)	2.17 to 3.50	**0.19**	0.44	0.86	.00 to .99	0.61	-.38 to .96
Communication	2.54 (.37)	1.72 to 3.15	0.19	0.44	0.86	.00 to .98	0.68	.01 to .92
Mission	3.01 (.18)	2.72 to 3.40	0.00	0.00	0.92	.51 to .99	0.73	-.75 to 1.00
Stress	2.75 (.35)	2.19 to 3.50	0.17	0.42	0.83	.00 to .97	0.64	.13 to 1.00
Total	2.78 (.18)	2.38 to 3.07	0.16	0.39	0.93	.00 to 1.00	0.67	-.06 to .90
Total without Mission	2.65 (.25)	1.99 to 3.13	**0.18**	0.44	0.93	.00 to 1.00	0.67	-.03 to .90

**Bold** indicates variance explained by level 2 (supervisors) was significantly different from 0 using likelihood ratio test, *p* < .05

#### Attitudes

Supervisors completed the Evidence-Base Practice Attitudes Scale 50-item version (EBPAS-50; Aarons et al., 2021) from August 2017 to January 2019 as part of a battery of surveys delivered during study training events, site visits to collect data, and follow up with individual providers. For the purposes of the current study, the 15 items representing the original EBPAS scale were examined (Aarons, 2004), given that (a) the EBPAS-15 total score is commonly used to represent global attitudes towards evidence-based practices and (b) previous studies have established United States National norms for the EBPAS-15, thus adding helpful context for scores observed in the current study (Aarons et al., 2010). Each item was scored on a five-point scale from 0 (not at all) to 4 (to a very great extent), with lower scores indicative of less favorable attitudes towards evidence-based practices. The total score was calculated using the mean of the 15 items resulting in scores that could range from 0 to 4. In Aaron’s (2004) study, the reliability estimate for the total scale was *a* = 0.77. In the current study, the reliability estimate for the total scale was *a* = 0.75. Records were removed if more than three items (20%) were missing. Missing values were computed using person mean substitution (Downey & King, 1998; Hawthorne & Elliott, 2005). In the current dataset, 22% (*n* = 5) respondents had any missing data and 14% of participants (*n* = 3) had more than three missing items. Supervisor EBPAS-15 scores ranged from 2.47 to 3.80 with a mean of 3.25 (*SD* = 0.42).

#### Communication

In addition to reflecting on communication at the organizational level via the TCU-ORG, therapists were also asked to reflect on communication at supervisory team level via the same set of Communication subscale items with different instructions: “Please consider your supervision team when answering the following questions again.” Referent-shift consensus items refer to items that are worded such to prompt respondents to answer questions in relation to the same level of aggregation, in this specific case, to answer questions in relation to the supervisory team to which they belong (Chan, 1998). Missing data for this scale were handled the same way as missing data from the broader TCU-ORG scale; 4% of therapists (*n* = 3) were missing more than half of the items on the subscale and their scores could not be calculated.

### Data Analysis

Analyses were conducted using R (R Core Team, 2021) using the packages nlme (Pinheiro et al., 2022), psych (Revelle, 2022), and multilevel (Bliese, 2016).

#### Perceptions of Climate

The first aim of the study was to determine the extent to which therapists’ perceptions of organizational climate were shared within supervisory teams and unique from other supervisory teams. Agreement indices capture the extent to which scores generated by different respondents are equivalent in terms of absolute value (e.g., both Therapist A and Therapist B from Supervisory Team 1 rate climate as a “3” on a five-point Likert scale; Bliese, 2000). Although historically the r_wg(j)_ index has been used to characterize agreement for multi-item scales, it has several limitations (Brown & Hauenstein, 2005). First, r_wg(j)_ uses a uniform null distribution (i.e., assumes all Likert scale points are equally likely to be selected) for comparison when estimating agreement. This is problematic because it does not account for rater bias (e.g., tending to use the higher end of the scale because of social desirability; Bliese, 2000). In addition, the r_wg(j)_ index is influenced by sample size and number of points on the Likert scale (Brown & Hauenstein, 2005). Some researchers have recommended use of other null response distributions (e.g., Beimann et al., 2012) to overcome the first limitation. However, Brown and Hauenstein (2005) argue that it is challenging to determine which null distribution is valid for a given scale and instead propose use of the a_wg,_ which addresses the above limitations. In the current study, both the r_wg(j)_ and the a_wg_, were calculated, enabling readers to compare this study’s findings with previous literature referenced in the introduction (r_wg(j)_; e.g., Aarons & Sawitzky, 2006; Glisson & Green, 2011) while also generating the most accurate index of agreement per best-practice recommendations from the organizational management literature (a_wg_).

Although agreement indices are useful for determining equivalence of ratings given by therapists in the same supervisory team, they do not provide information about the extent to which therapists from different supervisory teams are distinct from each other. This means that a high value observed for a_wg_ could be the result of high agreement within groups or high agreement across the entire sample. For this reason, it is important to contextualize agreement indices by pairing them with reliability indices. Reliability indices capture relative consistency among respondents (e.g., In Supervisory Team 1, Therapist A rates items one through three as “2”, “3”, “4” and Therapist B rates items one through three as “3”, “4”, and “5”; Bliese, 2000). Two types of interclass correlation coefficients (ICC (1) and ICC (2)) were calculated using multilevel models (therapists nested within supervisors) with random intercepts and no predictors for each climate subscale (i.e., Mission, Cohesion, Autonomy, Stress, and Change) and total score. ICC (1) is a measure of the proportion of total variance explained by group membership and was calculated by dividing between-group variance by total variance. A likelihood ratio test, which compares a model with random intercepts and no predictors to a model with no random intercepts and no predictors was used to determine if ICC (1) values were significantly different from zero. ICC (2) is a measure of the reliability of group means (e.g., mean ratings reliably distinguish groups; LeBreton & Senter, 2008) and was calculated by dividing the difference of between and within group variance by between group variances. A lower ICC (2) can occur when members across groups provide similar ratings.

#### Predictors of Climate

The second aim of the study was to investigate the association between candidate supervisor characteristics and therapist perceptions of climate subscales and climate total score. Given that therapists were nested within supervisors, multilevel models with random intercepts were used to assess whether supervisor attitudes towards evidence-based practices and supervisor communication predicted therapist perceptions of organizational climate. Site (Los Angeles, CA; Santee Wateree, SC; Pee Dee, SC) was included as a covariate given known differences in context including frequency and amount of time spent in supervision, case load sizes, and policies supporting EBT use (i.e., Prevention and Early Intervention Plan, Los Angeles Department of Mental Health, 2022). Assumptions of multilevel modeling were met, including normality of residuals for both level 1 and level 2 predictors, homoscedasticity, and assumptions of linearity.

## Results

### Perceptions of Climate

We first evaluated the extent to which therapists’ perceptions of organizational climate were shared within supervisory team and unique from other supervisory teams using two agreement indices (r_wg(j)_, and a_wg_) and two reliability indices (ICC (1) and ICC (2)). Values ranged from mean r_wg(j)_ 0.61 for Cohesion to mean rating r_wg(j)_ 0.74 for Autonomy and Change. Values ranged from mean a_wg_ 0.61 for Cohesion to mean a_wg_ 0.74 for Autonomy. Agreement values greater than or equal to 0.51 represent “moderate agreement” and values greater than or equal to 0.71 represent “strong agreement” (LeBreton & Senter, 2008). See Table 2 for mean and range and Table 3 for a_wg_ indices for each supervisory team. Table 2 also includes the ICC (1) and ICC (2) for each TCU-ORG subscale and total score. ICC (1) values ranged from 0.00 for the subscale Mission to 0.38 for the subscale Autonomy. Likelihood ratio tests indicated that ICC (1) values for Autonomy (ICC (1) = 0.38) and Cohesion (ICC (1) = 0.19) were significantly different from zero. Because the ICC (1) was so low for the Mission subscale, the TCU total score and ICC (1) was recalculated without it. This resulted in a new ICC (1) of 0.18 which was significantly different than zero as indicated by the likelihood ratio test. ICC (2) values ranged from 0.00 for the subscale Mission to 0.66 for the subscale Autonomy. The ICC (2) value for Cohesion was 0.44. The collective findings suggest that therapists on the same supervisory teams had similar perceptions of organizational climate that were also distinct from those of therapists on different supervisory teams for the subscales Autonomy, Cohesion, and TCU total score with Mission removed.

**Table 3:** Agreement Index (a_wg_) by Supervisory Team

Supervisory Team	Therapists (*n*)	Autonomy	Cohesion	Communication	Change	Stress	Mission	Total	Total without Mission
1	5	**0.72**	0.39	**0.76**	0.89	**0.76**	**0.71**	0.68	0.68
2	6	**0.85**	0.65	**0.82**	0.58	0.60	**0.83**	**0.73**	**0.71**
3	1	N/A	N/A	N/A	N/A	N/A	N/A	N/A	N/A
4	4	**0.92**	**0.91**	0.65	**0.77**	0.52	0.76	**0.76**	**0.76**
5	4	**0.71**	0.68	0.76	0.67	0.51	**0.81**	0.70	0.66
6	5	0.36	0.60	0.63	**0.76**	0.60	**0.73**	0.57	0.55
7	2	**0.78**	**0.78**	**0.78**	**0.78**	**0.90**	**0.78**	**0.78**	**0.78**
8	3	**0.78**	**0.93**	**0.88**	**0.96**	**0.85**	**0.88**	**0.78**	**0.78**
9	4	**0.76**	**0.93**	0.53	**0.92**	**0.79**	**0.81**	**0.79**	**0.80**
10	2	**0.78**	**0.78**	**0.89**	**0.78**	**1.00**	**0.78**	**0.82**	**0.83**
11	5	**0.91**	0.55	**0.72**	**0.73**	**0.83**	**0.86**	**0.75**	**0.73**
12	4	**0.90**	N/A	**0.78**	**0.80**	0.67	**1.00**	**0.83**	**0.79**
13	6	**0.81**	0.46	**0.75**	0.69	0.68	**0.81**	0.70	0.68
14	3	**0.73**	0.30	0.63	0.70	0.33	**0.95**	0.65	0.59
15	6	**0.86**	0.55	0.62	**0.71**	0.63	**0.74**	0.64	0.62
16	3	**0.80**	**0.96**	**0.92**	0.95	**0.80**	**0.95**	**0.90**	**0.90**
17	6	0.69	0.70	0.59	0.67	0.42	0.59	0.62	0.63
18	1	N/A	N/A	N/A	N/A	N/A	N/A	N/A	N/A
19	4	0.22	-0.38	0.01	0.08	0.13	-0.75	-0.06	-0.03
20	7	0.68	0.60	0.51	0.69	0.45	**0.83**	0.66	0.62
21	1	N/A	N/A	N/A	N/A	N/A	N/A	N/A	N/A
22	1	N/A	N/A	N/A	N/A	N/A	N/A	N/A	N/A

**Bold** indicates “strong agreement”; a_wg_ index cannot be calculated for groups of one

### Predictors of Climate

We then investigated the association between candidate supervisory characteristics (supervisor attitudes towards evidence-based practices measured by the supervisor self-report EBPAS and therapist perceptions of supervisor communication) and therapist perceptions of climate subscales. Cohesion, Autonomy, and total climate score (without Mission) were the three scales for which a significant amount of variance was attributable to supervisory team membership. Because the present study centers on supervisors’ influence on shared group perceptions of climate, only these scales were used as outcome variables in Aim 2.

Autonomy was regressed on site (Pee Dee, SC; Santee Wateree, SC; Los Angeles, CA), supervisor attitudes toward evidence-based practices, and therapist rated supervisor communication in a multi-level model with therapists nested within supervisors. In this model, site was a significant predictor of Autonomy such that therapists in Pee Dee and Santee Wateree rated Autonomy significantly higher than therapists in Los Angeles, CA (Pee Dee *b* = 0.35, *p* < 0.01; Santee Wateree *b* = 0.25, *p* < 0.05), over and above the effect of supervisor attitudes towards evidence-based practices and therapist rated supervisor communication. Therapist rated supervisor communication was a significant predictor of Autonomy such that for every one unit increase in therapist rated supervisor communication, Autonomy ratings increased by 0.34 (*b* = 0.34, *p* < 0.01) over and above the effect of supervisor attitudes towards evidence-based practices and site. In other words, therapists perceived more freedom and latitude in doing their jobs when supervisors engaged in clear, bidirectional communication that kept therapists informed. Supervisor attitudes towards evidence-based practices were not a significant predictor of Autonomy (*b* = -0.04, *p* = 0.74; see Table 4).

**Table 4:** Predictors of Therapist Perception of Autonomy in Organization

Factor	*b*	*SE*	*p*
Intercept	1.81	0.37	0.00
EBPAS-15	-0.04	0.11	0.74
Communication	0.34	0.05	0.00
*Site*			
Pee Dee	0.35	0.10	0.01
Santee Wateree	0.25	0.11	0.03
Variance Components	Variance	*SD*	
Residual	0.04	0.20	
Intercept	0.02	0.13	

Therapists (*n* = 76), supervisors (*n* = 19); Los Angeles was the reference group for Site

Cohesion was regressed on site (Pee Dee, SC; Santee Wateree, SC; Los Angeles, CA), supervisor attitudes toward evidence-based practices, and therapist rated supervisor communication in a multi-level model with therapists nested within supervisors. In this model, therapist rated supervisor communication was a significant predictor of Cohesion such that for every one unit increase in therapist rated supervisor communication, Cohesion ratings increased by 0.70 (*b* = 0.70, *p* < 0.01) over and above the effect of supervisor attitudes towards evidence-based practices and site. In other words, therapists perceived higher workgroup cooperation and teamwork when supervisor communication was strong. Supervisor attitudes towards evidence-based practices (*b* = 0.15, *p* = 0.51) and site (Pee Dee *b* = 0.06, *p* = 0.80; Santee Wateree *b* = 0.11, *p* = 0.61) were not significant predictors of Cohesion (see Table 5).

**Table 5:** Predictors of Therapist Perception of Cohesion in Organization

Factor	*b*	*SE*	*p*
Intercept	0.57	0.73	0.44
EBPAS-15	0.15	0.22	0.51
Communication	0.70	0.10	0.00
*Site*			
Pee Dee	0.06	0.22	0.80
Santee Wateree	0.11	0.21	0.61
Variance Components	Variance	*SD*	
Residual	0.17	0.41	
Intercept	0.06	0.25	

Therapists (*n* = 76), supervisors (*n* = 19); Los Angeles was the reference group for Site

Total Climate score excluding the Mission subscale was regressed on site (Pee Dee, SC; Santee Wateree, SC; Los Angeles, CA), supervisor attitudes toward evidence-based practices and therapist rated supervisor communication in a multi-level model with therapists nested within supervisors. In this model, therapist rated supervisor communication was a significant predictor of Total Climate score such that for every one unit increase in therapist rated supervisor communication, Total Climate score ratings increased by 0.59 (*b* = 0.59, *p* < 0.01) over and above the effect of supervisor attitudes towards evidence-based practices and site, suggesting that therapist perception of supervisor communication had generalized positive effects on perception of climate overall. Therapists in the site, Pee Dee, SC, had significantly higher ratings of climate compared with Los Angeles, CA therapists (*b* = 0.21, *p* < 0.05). Therapists in the site, Santee-Wateree, SC, did not have significantly higher ratings compared with Los Angeles, CA therapists (*b* = 0.16, *p* = 0.08). Supervisor attitudes towards evidence-based practices (*b* = 0.15, *p* = 0.51) was not a significant predictor of Total Climate score (see Table 6). Therapist age and years of clinical experience were investigated as possible covariates but were not associated with any of the outcomes of interest and are therefore not reported here.

**Table 6:** Predictors of Therapist Perception of Organizational Climate

Factor	*b*	*SE*	*p*
Intercept	0.93	0.30	0.00
EBPAS-15	0.03	0.09	0.73
Communication	0.59	0.06	0.00
*Site*			
Pee Dee	0.21	0.09	0.03
Santee Wateree	0.16	0.09	0.08
Variance Components	Variance	*SD*	
Residual	0.06	0.03	
Intercept	0.00	0.24	

Therapists (*n* = 76), supervisors (*n* = 19); Los Angeles was the reference group for Site

## Discussion

The main goal of the present study was to explore the role of clinical supervisors as drivers of therapist perceptions of organizational climate. Specifically, this study explored perceptions of organizational climate among therapists nested in supervisory teams and investigated candidate predictors of therapists’ perceptions of climate.

### Shared Perceptions of Organizational Climate

Consistent with the hypothesis that therapists in the same supervisory team will have more shared perceptions of organizational climate compared with therapists in different supervisory teams, team membership accounted for a significant amount of variance for Autonomy, Cohesion, and total climate score (removing the subscale, Mission; as indicated by ICC(1)). Interestingly, results also suggest that subdomains of organizational climate may be differentially influenced by group membership. Taking the most extreme example, supervisory team explained almost no variance in therapists’ perception of organizational Mission (ICC (1)). However, agreement about Mission for therapists across all groups was generally strong (as indicated by a_wg_). Conversely, supervisory team explained a large, statistically significant amount of variance in organizational Autonomy and Cohesion. Therapists in the same group also tended to agree about organizational Autonomy and Cohesion and mean ratings for each group were somewhat reliable when distinguishing between groups (as indicated by ICC(2)). This pattern suggests that although therapists, across all supervisory groups, had a consistent shared understanding of organizational mission, their perceptions of day-to-day interpersonal relationships with other employees (i.e., cohesion) and independence to conduct their work (i.e., autonomy) were influenced by their direct supervisor.

This pattern maps onto Birken et al.’s (2012) theory on the role of middle managers in the implementation of innovations. Specifically, organizational mission and priorities are expressed at the upper levels of the organization and would therefore be expected to remain stable regardless of supervisory team. In turn, middle managers translate the mission and priorities into day-to day interactions and activities on their teams thus impacting a sense of cohesion and autonomy (Bunger et al., 2019). It is especially notable that supervisory team was associated with almost 40% of the variability in perceptions of autonomy. Although there are some conflicting findings in the literature, generally, autonomy has been associated with an increased sense of personal achievement and reduced emotional exhaustion at work, both important factors for preventing professional burnout (Yang & Hayes, 2020).

Supervision has been described as serving three key functions, one of which is characterized as restorative, i.e., support of supervisee emotional wellbeing (Milne, 2007). With this in mind, it is surprising that supervisory team was not associated with significant variability in therapist ratings of stress. It is possible that the influence of organizational factors outside of the control of the supervisor such as productivity standards and caseload size, were so prominent as to negate any possible impact of the supervisor (Franco, 2016). It may also be that the focus of supervision tended not to include restorative activities. The latter explanation is consistent with previous literature examining activities in supervision-as-usual in the context of community mental health serving youth. In their study, Bailin et al. (2018) found the most common supervision activities were administrative tasks and praise (both occurring in almost 90% of supervision sessions coded for about a quarter of the time in supervision). However, an explicit focus on supervisee wellbeing occurred in less than half of sessions and tended to take up less than 5% of the time in supervision. However, it is important to acknowledge that it is possible that other activities in supervision decrease therapist stress beyond an explicit focus on wellbeing.

### Supervisor Characteristics as Predictors of Organizational Climate

Supervisor attitudes toward evidence-based practices did not predict therapist perceptions of overall climate, cohesion, or autonomy. This finding was not consistent with the study’s second hypothesis that supervisor attitudes would be significantly and positively associated with therapist perceptions of organizational climate. This is surprising given past research that found an association between therapist attitudes and aspects of organizational climate (Aarons & Sawitzky, 2006; Aarons & Sommerfeld, 2012). Based on these previous findings, one might anticipate that, because supervisors have greater organizational power and influence, supervisor attitudes would serve as a powerful driver of organizational climate. However, attitudes toward evidence-based practices from supervisors in the current sample were higher and more homogenous than national norms (present study: *M* = 3.25, *SD* = 0.42; national norms: *M* = 2.73, *SD* = 0.49; Aarons et al., 2010). This pattern may be reflective of the growing national movement to promote the use of evidence-based practices and may further reflect the efforts in Los Angeles County specifically. The Los Angeles Country Prevention and Early Intervention Plan was approved by the state in 2009 and provides reimbursement for the delivery of evidence-based practices and sponsored trainings in six treatment programs for community mental health organizations (Los Angeles Department of Mental Health, 2022). It is therefore possible that a sample with greater variability and more negative views of evidence-based practices may have yielded different results.

Consistent with the study’s third hypothesis, therapist perceptions of supervisor communication significantly predicted overall climate, cohesion, and autonomy. It is possible therapists were aware of the overarching organizational mission but their direct work experience (cohesion among employees and autonomy to conduct their work) was influenced by the direct communication of the supervisor. This finding, again, aligns with emerging evidence that middle managers shape their employees’ experience through communication strategies (Birken et al., 2012; Bunger et al., 2019). When investigating types and methods of communication that may be particularly important for a positive organizational climate, Bunger et al. (2019)’s study emphasized the importance communication that serves to provide support for the use of new interventions and provides persuasive information about new interventions. In a qualitative study focused on the implementation of measurement-based care, results also emphasized the importance of communication that uses multiple channels and media (i.e., individual, group and electronic communication) and has opportunities for bi-directional engagement (clarifying questions, requests for additional information, ability to offer input; Albright et al., 2022).

### Limitations

This study had several limitations. First, the level-two (supervisor) sample size was small thus increasing the chances of type 1 error for analyses using supervisor level predictors (McNeish & Stapleton, 2016). Though it is possible that a larger sample of supervisors may have yielded different results, in simulation studies, level-two samples as low as 15 yielded unbiased estimates of fixed effects. Conversely, level-two samples of 30 or higher may be necessary for unbiased estimates of fixed-effect standard errors. Samples smaller than 30, therefore, increase the chances of type 1 error (McNeish & Stapleton, 2016). However, despite the potential for inflated *p*-values, supervisor attitudes of EBTs were not found to be a predictor of therapist perception of climate, perhaps increasing confidence in this result.

Second, therapist perceptions of supervisor communication were collected concurrent with perceptions of broader organizational climate (of note, participants first completed measure in response to the organization and then completed the measure in response to their supervisor on a separate page). The association between supervisor communication and climate scales, may therefore, be a result of a response bias (e.g., tendency to respond positively across all scales due to a halo effect). Future research might consider exploring this association further by using objective measures of supervisor communication strategies. However, a therapist’s impression of how their supervisor communicates (rather than the “true” quality of communication) may be uniquely important for informing how therapists feel about the organization they work in.

The subscale Autonomy exhibited low internal consistency. For this reason, current results should be interpreted with caution and attempts should be made to replicate to increase confidence in results. Internal consistency for this subscale was low relative to other subscales in other studies as well (Lehman et al., 2002; *a* = 0.57). Considering observed internal consistency in the present study and creating study, researchers may consider using other measures of organizational autonomy. For example, this construct is captured in the Organizational Climate Measure (also called the “Autonomy” subscale *a* = 0.67, Patterson et al., 2005).

Finally, organizations are often comprised of many units and subunits, and it is challenging to know which units are impacting an employees’ experience of their work environment (e.g., care network, hospitals, department, unit, shift). Although this study dealt specifically with the supervision level and accounted for site level through inclusion as a covariate, other levels of possible importance were not assessed. For example, in addition to the site level, therapists (*n* = 45) and their supervisors (*n* = 12) in CA tend to work in specific “neighborhoods” comprised of several schools. The sites in SC (Pee Dee and Santee Wateree) were much smaller, and therapists (*n* = 41) and supervisors (*n* = 10) tend to move between multiple schools in the area making the influence of a school or neighborhood level less likely. However, given that CA therapists comprised approximately half of the sample, the grouping seen in Aim 1 may have been driven by some feature of the neighborhood instead of or in addition to the characteristics of the supervisor.

### Future Directions

Availability, Responsiveness, and Continuity (ARC) is an example of an intervention that has been shown to improve climate and reduce staff turnover by intervening at the full organizational as well as the interorganizational level (i.e., collections of organizations and relevant stakeholders in the community; Glisson & Schoenwald, 2005; Glisson et al., 2006;). Although ARC uses a macro-level approach, it is possible that more micro-levels of the organization may contribute to organizational climate and be more pragmatic for intervention depending on the size and composition of the organization. The findings that therapist perceptions of the communication on their supervisory team were associated with perception of organizational climate overall, cohesion, and autonomy provide evidence that supervisors play an important role in shaping perceptions of climate and may represent an additional avenue for intervention. To better disentangle the role of the supervisor from aspects of the organization at other levels, future research should further explore the association between climate and supervisory level characteristics such as leadership style, working alliance, and supervisory behaviors (Aarons & Sommerfeld, 2012; Dorsey et al., 2017). Future studies might also identify and test strategies for increasing the ability of the supervisor to have an impact on organizational climate such as examining the impact of additional protected time for supervision or training in specific supervisory behaviors that foster a positive perception of the organization. For example, prior work has shown an association between confidence in ability to use evidence-based treatments (Kim et al., 2018) and support to use evidence-based treatment (Sripada et al., 2024) with reduced burnout. Perhaps supervision behaviors that have been shown to promote therapists to use EBTs are also linked to an improved perception of organizational climate.

## Conclusions

The present study capitalized on prior research and methodology from the organizational management literature to explore the role of supervisors in shaping therapists experience of their work environment. Results build upon past research describing the key role of middle managers as intermediaries between upper-level management and front-line workers, particularly in terms of perceptions of cohesion and autonomy. Further elucidating the role of supervisors in mental health organizations will be important for maximizing workforce capacity to address the substantial and growing demand for mental health treatment.
